# Tick-borne diseases of bovines in Pakistan: major scope for future research and improved control

**DOI:** 10.1186/s13071-015-0894-2

**Published:** 2015-05-22

**Authors:** Abdul Jabbar, Tariq Abbas, Zia-ud-Din Sandhu, Hafiz A Saddiqi, Muhammad F Qamar, Robin B Gasser

**Affiliations:** Faculty of Veterinary and Agricultural Sciences, The University of Melbourne, Werribee, Victoria Australia; University College of Veterinary & Animal Sciences, The Islamia University of Bahawalpur, Punjab, Pakistan; Department of Parasitology, University of Agriculture, Faisalabad, Punjab Pakistan; Department of Zoology, Government College University, Faisalabad, Punjab Pakistan; College of Veterinary and Animal Sciences, University College of Veterinary and Animal Sciences, Jhang, Punjab Pakistan

**Keywords:** Tick-borne diseases, *Theileria*, *Babesia*, *Anaplasma*, Cattle, Water buffalo, Pakistan

## Abstract

Ticks and tick-borne diseases (TBDs) affect the productivity of bovines in tropical and subtropical regions of the world, leading to a significant adverse impact on the livelihoods of resource-poor farming communities. Globally, four main TBDs, namely anaplasmosis, babesiosis, theileriosis, and cowdriosis (heartwater) affect bovines, and the former three are of major economic importance in bovines in Pakistan. Given that the livestock sector has become an integral part of Pakistan’s economy and a large number of dairy cattle are being imported into the country, in order to meet an increasing demand of milk and milk products, it is timely to review current status of bovine TBDs in Pakistan and to identify gaps in the knowledge of TBDs and their control. Although there has been a recent increase in the number of studies of TBDs in this country, information on their prevalence, distribution, tick vectors, and control is limited. This article provides a brief background on key bovine TBDs and ticks and reviews the current status of bovine TBDs in Pakistan to identify gaps in knowledge and understanding of these diseases, propose areas for future research and draw attention to the need for improved tools for the diagnosis and control of TBDs in this country.

## Introduction

Livestock plays a pivotal role in Pakistan’s economy by uplifting the socioeconomic conditions of resource-poor farming communities and alleviating poverty. The livestock sector in Pakistan is represented mainly by small farm holders to meet the needs of nutrients and proteins, food security, and income. In the financial year 2013/2014, the livestock sector contributed 11.8 % to the Gross Domestic Product (GDP) of Pakistan; its share in the value of all agricultural commodities was 55.9 % [1]. In the livestock sector, water buffaloes, and cattle with an estimated population size of 35–40 million, are the main milk-producing animals, and yielding approximately 18,000–31,000 million tons of milk [1]. Based on location and herd size, dairies in Pakistan are classified into four systems, including *smallholder subsistence* (milk produced to meet household needs), *smallholder market-oriented* (milk produced for home use with small, but regular surpluses for sale); *rural commercial* (larger herds of >40 animals, well organised, with direct links to milk processing plants); and *peri-urban* (gowalas; animal husbandrists in the outskirts of cities, with herd size of ~ 20 animals, selling into urban areas) [2, 3].

Based on climate, water availability, land use, and physiography, Pakistan is divided into ten agro-ecological zones, which influence temporal, and spatial patterns of livestock diseases. Being located in a subtropical zone (30° N, 70° E) within South Asia, most parts of Pakistan offer favourable environmental conditions for ticks, which can infest a variety of hosts and transmit diseases to humans, livestock, and companion animals. Ticks and tick-borne diseases (TBDs) cause substantial economic losses in bovines, particularly in tropical, and subtropical regions, where 80 % of the world’s total cattle population occurs [4], and can significantly affect the livelihoods of resource-poor farming communities due to lower productivity of both beef and dairy cattle in these regions [5, 6].

Theileriosis (caused by *Theileria annulata*), babesiosis (*Babesia bovis*, *B. bigemina,* and *B. divergens*) and anaplasmosis (*Anaplasma marginale* and *A. centrale*) have been reported to affect both water buffaloes (*Bubalus bubalis*) and cattle (*Bos indicus* and *Bos taurus*) in Pakistan [7–9]. Given the relatively rapid expansion of the dairy industry and the increased importation of high milk-producing dairy cattle (e.g., Holstein, and Friesian) from overseas countries to replace and/or improve indigenous local breeds of cattle, it has become crucial to assess the status of TBDs in indigenous and exotic breeds of cattle and water buffaloes, as exotic breeds are usually highly susceptible to TBDs [10]. The purpose of this article is (i) to provide a brief background on key bovine TBDs and ticks, (ii) to review the current state of knowledge of bovine TBDs in Pakistan, (iii) to identify gaps in the knowledge and understanding of these diseases, and (iv) to propose areas for future research, focusing on developing improved approaches for their diagnosis and control in this country.

### Background on TBDs and tick vectors relevant to the context in Pakistan

Principal bovine TBDs, the main pathogens involved, their distribution, and tick vectors are given in Table 1. In addition, the status of these diseases and ticks in their transmission in Pakistan are listed. The following section provides an account of the three key bovine TBDs in Pakistan.

**Table 1:** Major tick-borne diseases (TBDs) of bovines: principal pathogens, principal hosts, principal vectors, and distribution. Current status of the main bovine TBDs and potential vectors in Pakistan are also provided.

Tick-borne disease (TBDs)	Principal pathogens	Principal host (s)	Principal ticks vectors	Distribution	Bovine TBDs reported from Pakistan	Possible tick vectors in Pakistan
Theileriosis	*Theileria annulata*	Cattle, Asian buffalo	*Hyalomma detritum detritum*	Southern Europe, North Africa, Middle East, Sudan, central Asia, and Indian subcontinent,	Yes	H. anatolicum anatolicum,
			*H. anatolicum*			
			*H. dromedarii*			H. dromedarii,
			*H. lusitanicum*			
						H. marginatum marginatum
	*T. parva*	Cattle, African buffalo	*Rhipicephalus appendiculatus, R. zambeziensis*	Eastern, central and southern parts of Africa	No	Not applicable (NA)
	*T. orientalis*	Cattle, Asian buffalo	*Haemaphysalis longicornis*	Cosmopolitan	No	NA
	*T. mutans*	Cattle, African buffalo	*Amblyomma* spp.	Sub-Saharan Africa	No	NA
	*T. velifera*	Cattle, African buffalo	*Amblyomma* spp.	Sub-Saharan Africa	No	NA
	*T. taurotragi*	Cattle, Eland antelope	*R. appendiculatus*	East and southern Africa	No	NA
Babesiosis	*Babesia bigemina*	Cattle, buffalo	*R. (B.) microplus*	Tropical and subtropical regions	Yes	*R. (B.) microplus*
	*B. bovis*	Cattle, buffalo	*R. (B.) microplus*	Tropical and subtropical regions	Yes	*R. (B.) microplus*
	*B. divergens*	Cattle	*Ixodes ricinus*	Europe and North Africa	No	NA
	*B. orientalis*	Buffalo	*Rhipicephalus* spp.	Asia	No	NA
Anaplasmosis	*Anaplasma marginale*	Cattle, domestic buffalo	*Rhipicephalus (Boophilus) microplus, R. (B.) decoloratus, R. (B.) annulatus, R. bursa, R. simus, R. evertsi*	Tropical, subtropical and even temperate regions	Yes	*R. (B.) microplus*
						*Hyalomma* spp.
	*A. centrale*	Cattle	As above	Tropical and subtropical regions	Yes	As above
	*A. (Ehrlichia) bovis*	Cattle, domestic buffalo	*Amblyomma* spp., *Hyalomma* spp., *Rhipicephalus* spp.	Africa, Asia, and South America	No	NA
	*A. (Ehrlichia) phagocytophilum* ^*a*^	Cattle, small ruminants, horses	*Ixodes* spp.	Europe and North America	No	NA

^a^Zoonotic importance; Source: [7, 10, 18, 20, 29, 31]

#### Theileriosis

Theileriosis is a disease caused by intracellular protozoan parasites of the genus *Theileria* (Apicomplexa: Piroplasmida; Theileriidae), transmitted by ixodid ticks. Usually, the geographical distribution of *Theileria* species is restricted to tropical and subtropical regions, where suitable tick vectors occur. *Theileria* species infect primarily wild and domestic ruminants, and cause economically significant diseases in cattle, sheep, and goats. For instance, *T. annulata,* and *T. parva* (the causative agents of tropical or Mediterranean and East Coast Fevers, respectively) are known to be the most pathogenic species in bovines, whereas other species, such as *T. mutans*, *T. taurotragi,* and members of the *T. orientalis* complex, often cause asymptomatic infections in this host group [11–13]. Depending on the type of theileriosis, a number of hard ticks belonging to the genera *Amblyomma*, *Haemaphysalis*, *Hyalomma,* and *Rhipicephalus* can transmit theilerioses (see Table 1) [14]. *T. annulata* causes a severe, potentially fatal disease in cattle, leading to significant economic losses in endemic countries in Africa and Asia, and is mainly transmitted by ticks of the genus *Hyalomma* [14]. In general, tropical theileriosis is more severe in exotic and cross-bred cattle (*Bos taurus*) than indigenous animals (e.g., *Bos indicus*) [10]. For example, the disease became significant in India when a program was launched to increase milk production by introducing exotic breeds. Mostly, the disease occurs in its subclinical form, leading to significant economic losses; without treatment or control, case fatality rates can reach 80 % in exotic breeds, compared with ~ 20 % in indigenous breeds [15, 16]. Major clinical signs include pyrexia, enlargement of lymph nodes, particularly of those draining the site of attachment of ticks, tachycardia, tachypnea, nasal discharge, loss of weight, and condition, severe pulmonary distress due to oedema, and death in severe cases [14]. Tropical theileriosis can be treated with the specific anti-*Theileria* drugs, such as buparvaquone, and parvaquone [14].

#### Babesiosis

Babesiosis, caused by infection with intraerythrocytic protozoan parasites of the genus *Babesia* (Apicomplexa: Piroplasmida: Babesiidae), is one of the commonest infections of animals worldwide. In recent years, the disease has been gaining interest in human medicine due to emerging zoonotic infections [17]. Bovine babesioses can be caused by a number of *Babesia* species, but *B. bovis*, *B. bigemina,* and *B. divergens* are the most important species, both economically, and clinically, in both water buffaloes and cattle (see Table 1). These diseases occur in temperate, subtropical, and tropical regions of the world and affect more than a billion cattle globally [18]. Depending on the form of babesiosis, species of *Rhipicephalus,* and *Ixodes* are involved in the transmission to bovines (see Table 1) [18]. *B. bovis* is considered to be the most pathogenic species, followed by *B. bigemina,* and *B. divergens* [18]. The former two species of *Babesia* occur in the tropics and subtropics, whereas the latter is prevalent in Europe and also of zoonotic importance [18]. Clinical signs depend on virulence and pathogenic effects of a particular *Babesia* species, and host factors associated with disease include age, breed, and immune status [18]. Major signs include high fever, depression, anorexia, haemoglobinaemia, haemoglobinuria, icterus, abortion in pregnant cows, and death in severe cases [18]. *B. bovis* infection can also cause nervous and respiratory symptoms due to the sequestration and effects of infected erythrocytes in the capillary beds of vital internal organs [19]. For treatment and prophylaxis of bovine babesiosis, diminazine, and imidocarb are the only two drugs available [18].

#### Anaplasmosis

Anaplasmosis is a disease of domestic and wild ruminants, caused by obligate intraerythrocytic rickettsiae of the genus *Anaplasma* (Rickettsiales: Anaplasmatacea), and is distributed in tropical and subtropical regions of the world. In bovines, anaplasmosis is caused mainly by *A. marginale* and, to a lesser extent, by *A. centrale*. The disease is usually transmitted by ticks, but it can also be transmitted mechanically by biting flies or contaminated surgical instruments and/or needles [20]. Almost 20 tick species have been shown to transmit anaplasmosis experimentally [21], and the most important tick genera involved in the transmission of this disease are *Hyalomma* and *Rhipicephalus* species (see Table 1) [20]. Clinically, the disease is manifested in a number of forms, from subclinical to fatal, depending on the virulence of the species/strain of *Anaplasma*, susceptibility of the host, or concurrent infections. Major clinical signs include pyrexia, progressive anaemia, jaundice, anorexia, depression, reduced milk production, abortion in pregnant animals, and death, particularly in exotic breeds [20]. Anaplasmosis is difficult if not impossible to differentiate clinically from theileriosis and babesiosis. Animals recovering from this disease usually develop an asymptomatic carrier status [20]. The treatment of bovine anaplasmosis includes the parenteral administration of tetracyclines (chlortetracycline and oxytetracycline) or imidocarb dipropionate [20].

### Economic significance of ticks and TBDs

Ticks themselves also cause substantial economic losses in cattle by reducing productivity and fertility, and sometimes causing deaths [22]. Tick infestation reduces the productivity of cattle in a number of ways, including: (i) the direct effect of attachment and feeding (‘tick worry’), (ii) the injection of toxins, (iii) hide damage due to their bites, (iv) a reduction in weight gain due to the sucking of blood by female, adult ticks (e.g., *Rhipicephalus microplus*), (v) reduced milk production, and quality, and (vi) morbidity and mortality associated with the diseases that they transmit [6, 23–26]. Various studies have estimated losses caused by ticks and TBDs in cattle. For example, De Castro [4] estimated the global production economic losses caused by ticks and TBDs at about USD 14–19 billion per year. Recently, studies in Australia, and India have also estimated annual losses at USD 26 million [27] and 499 million [28], respectively.

### Current knowledge of bovine TBDs in Pakistan

In Pakistan theileriosis, babesiosis, and anaplasmosis are major infectious diseases of water buffaloes and cattle, and are caused by *T. annulata, B. bovis,* and *B. bigemina* as well as *A. marginale* and *A. centrale*, respectively [7–9]. These diseases are transmitted by ixodid ticks, and a large number of these ticks infest water buffaloes and cattle in Pakistan, including species of *Dermacentor*, *Haemaphysalis*, *Hyalomma,* and *Rhipicephalus* [7, 29–33] (see Table 1). A number of species of *Hyalomma* and *Rhipicephalus* are considered to be the predominant vectors of bovine theileriosis and babesiosis, respectively [30]. To date, only one study has shown the role of *Hyalomma* spp. in the transmission of *T. annulata* by the molecular detection of the parasite in ticks [32] (see Table 2), whereas the role of other ticks in the transmission of other bovine TBDs in Pakistan remains to be explored. In the following sections, we reviewed the peer-reviewed scientific literature on bovine TBDs in Pakistan which were accessible on the 31st of March 2015 through CABI Abstracts, PubMed, and national scientific journals.

**Table 2:** List of key studies of Theileria/theileriosis in Pakistan

Host (s)	Location (s)	Study population and sampling design	Sampling period	Detection method (s)	Percentage of test positive animals (proportion)		Reference
					Cattle	Buffaloes	
Cattle (Sahiwal x Holstein-Friesian)	Faisalabad	Animals suspected of theileirosis from the University farm	May-Jun 1982	Blood smear	100 (3/3)	NA	[39]
Buffaloes, cattle (local breeds)	Hyderabad	Healthy animals from local private dairy farms; convenient	Oct 1990 to Dec 1991	Blood smear	3 (3/100)	5 (5/100)	[40]
Cattle (Jersey, Sahiwal, Holstein-Friesian, cross–bred)	Faisalabad	Animals suspected of theileriosis from local private dairy farms; convenient	Mar 1993 to Sep 1998	Clinical signs and blood smear	79.5 (89/112)	NA	[38]
Buffaloes, cattle (local and exotic breeds)	Attock, Islamabad	Healthy animals were from public livestock farms; convenient	Sep 1999 to May 2001	Blood smear	0.98 (3/307)	0.6 (1/155)	[41]
Cattle (local breeds)	Peshawar	Healthy animals from local private dairy farms; convenient	2001	Blood smear	1.4 (4/285)	NA	[42]
Cattle (Friesian and Jersey)	Kasur	Healthy animals from a public livestock farm; convenient	Jul 2003 to Jun 2004	Blood smear	Holstein: 24 (48/200)	NA	[43]
					Jersey: 15 (30/200)		
Buffaloes	Lahore	Animals suspected of theileriosis from local private dairy farms; convenient	Jul to Sep 2003	Blood smear	NA	17.5 (107/600)	[44]
Friesian cattle	Kasur	Healthy animals from local private dairy farms; convenient	-	Blood smear,	^1^BS: 14 (14/100)	NA	[7]
				PCR	^2^PCR: 36 (36/100)		
Holstein-Friesian and Jersey cattle	Sahiwal	Healthy animals from local private dairy farms; convenient	Apr to Sep 2009	Blood smear	38.3 (115/300)	NA	[45]
Buffaloes, cattle (local breeds)	Bahawalnagar, Bhakar, Layyah, Multan, Muzaffar Garh, Vehari	Healthy animals from local private dairy farms; convenient	-	Blood smear,	BS: 3 (4/144); PCR: 19 (28/144)		[37]
				PCR			
Buffaloes	Karachi	Healthy animals from the Landhi Dairy Colony; convenient	Apr to Oct 2011	Blood smear	N/A	2 (2/100)	[46]
Cattle (local and exotic breeds)	Sargodha	Healthy animals from local private dairy farms; multi-stage cluster random	Aug 2008 to Jul 2009	Blood smear	6.7 (24/350)	N/A	[47]
					^3^Mixed: 2.6 (9/350)		
Cattle (local and exotic breeds)	Khushab, Rawalpindi, Sargodha	Healthy animals from local private dairy farms; convenient	Sep 2009 to Aug 2010	Blood smear	5.14 (54/1050)	N/A	[48]
Cattle (local breeds)	Kohat, Peshawar	Healthy animals from local private dairy farms; simple random	Nov 2010 to Feb 2011	Blood smear,	BS: 5.3 (5/95)	NA	[36]
				PCR	PCR: 33.7 (32/95)		
Ticks (*Hyalomma anatolicum*, *Hyalomma dromedarii*)	Faisalabad, Jhang, Khanewal	Healthy animals from local private dairy farms; convenient	Jul, Aug 2007	PCR	^4^ *Ha*: 50 (10/20), ^5^ *Hd*: 20 (4/20)*		[32]
Buffaloes, cattle (Sahiwal breed)	Okara, Sheikhupura	Healthy animals from local private dairy farms; convenient	-	PCR	66.1 (41/62)	50 (20/40)	[35]

^1^Blood smear; ^2^Polymerase chain reaction; ^3^Mixed infection of *T. annulata* with either *A. marginale* or *B. bigemina*; ^4^ *Hyalomma anatolicum*, ^5^ *Hyalomma dromedarii*; *host information was not provided; − denotes lack of information

#### Theileriosis

**Fig. 1:**
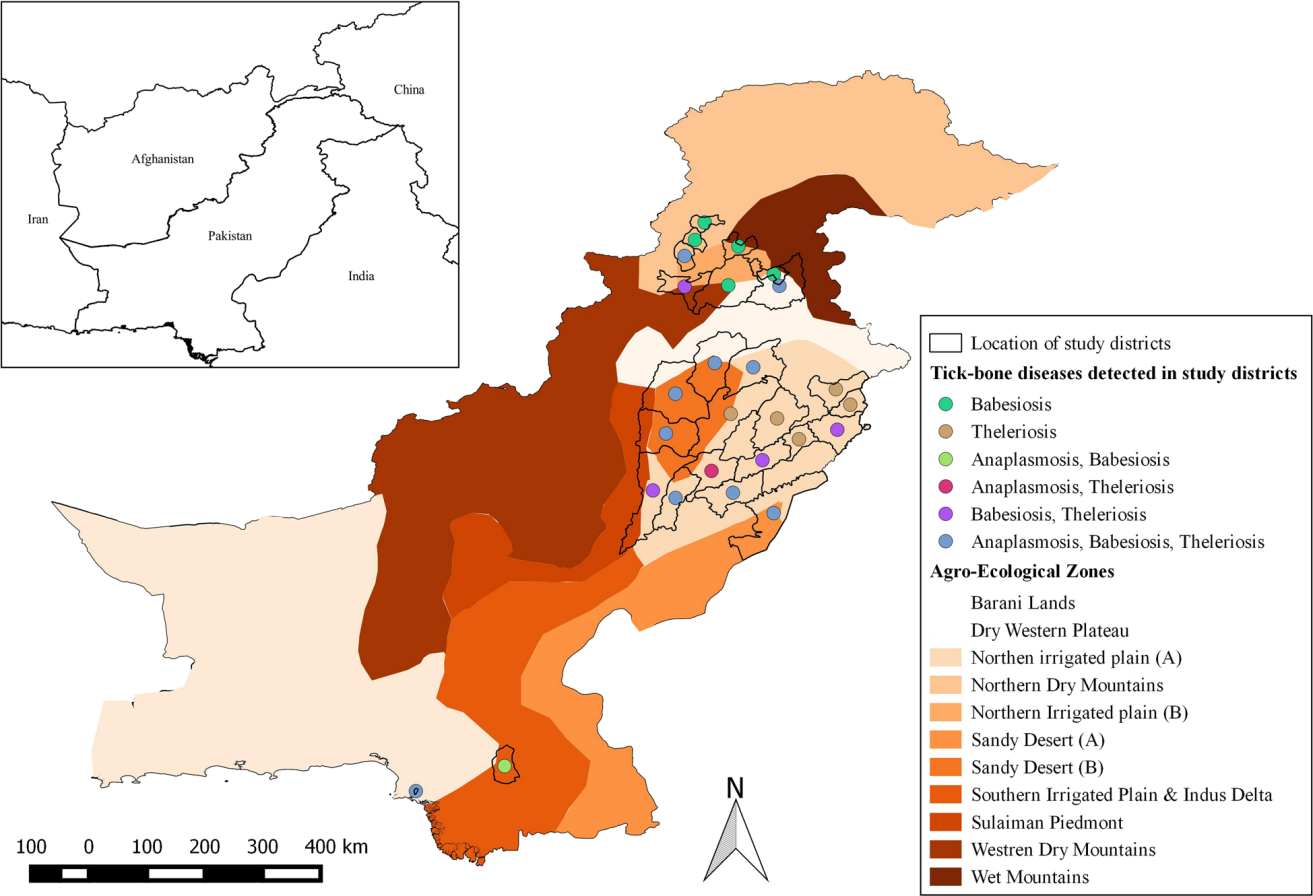
Map of Pakistan showing districts where prevalence studies of tick-borne diseases (shown in circles of different colours) have been conducted. The distinct colours on the map indicate various agro-ecological zones of Pakistan. The inset map shows the neighbouring countries of Pakistan.

The most studied bovine TBD in Pakistan is tropical theileriosis. To date, 16 studies have reported the prevalence of this disease (Table 2), with the majority of investigations being conducted in Punjab (*n*umber of districts studied = 19), followed by Khyber Pakhtunkhwa (*n* = 2) and Sindh (*n* = 2) provinces (Fig. 1; Table 2). In Pakistan, more studies of theileriosis have been undertaken in cattle (*n* = 13) than in water buffaloes (*n* = 6), and most of them investigated healthy animals located either on private dairy farms near the major veterinary research institutions or public livestock research stations (Table 2). Almost all of these studies used a convenient sampling strategy for the collection of blood samples, without defining criteria for the selection of a location or particular herds or number of animals to be tested, thus, likely leading to sampling bias and possible misrepresentation of the actual population in a district, an agro-ecological zone, or province. Using conventional diagnostic methods, the mean (± standard error of mean) prevalence rates of theileriosis in water buffaloes and cattle reported for Pakistan are estimated at 10.6 ± 3.5 % (range from 0.98 to 38.3 %) and 2.65 ± 0.9 % (0.6–5.0 %), respectively. However, higher prevalences were estimated when molecular diagnostic methods were used to test water buffaloes (34.5 ± 15.5 %; range 19.0–50.0 %) and cattle (38.7 ± 9.9; range from 19.0 to 66.1 %) [32, 34–37]. As a number of *Hyalomma* species are known to transmit *T. annulata*, a single study has thus far identified *T. annulata* from *H. anatolicum* (prevalence: 50.0 %; 10/20) and *H. dromedarii* (prevalence 20.0 %; 4/20) in Pakistan (Table 2, [32]). A number of risk factors have been predicted to be associated with tropical theileriosis in bovines in Pakistan. For example, Muhammad *et al.* [38] identified clinico-epidemiological factors to be associated with theileriosis in cross-bred cattle in Faisalabad and showed that young calves of cross-bred cattle were more susceptible to disease from February to November, with a disease peak occurring in June when tick activity was high.

#### Babesiosis

A total of 15 studies have reported bovine babesiosis from Pakistan, and most of them were conducted in Punjab (*n*umber of districts studied = 12), followed by Khyber Pakhtunkhwa (*n* = 3) and Sindh (*n* = 2) provinces (Fig. 1; Table 3). The majority (*n* = 14) of these investigations focused on exotic and local breeds of cattle, whereas only four studied the disease in water buffaloes (see Table 3). The mean (±standard error of mean) prevalence rates of babesiosis in water buffaloes and cattle reported for Pakistan are estimated at 7.5 ± 1.5 % (range: 0.65–29.0 %) and 5.7 ± 4.4 % (range: 1.0–23.1 %), respectively. Using a traditional diagnostic method (i.e., stained blood smear), the prevalence of babesiosis was considerably lower (1–18 %) than that obtained employing molecular diagnostic methods (up to 29 %) (Table 3; [49, 50]). Bovine babesiosis mostly occurs in exotic (susceptible) breeds of cattle during the hot and humid months (July to September). Like theileriosis, the precise prevalence of babesiosis in Pakistan is unknown, as there seems to have been a sampling bias as a consequence of the selection of animals, locations, and agro-ecological zones investigated. Although *R. microplus* is the main vector of babesiosis worldwide, to date, there has been no study to assess the role of this tick, other vectors, or proposed mechanical means of disease transmission.

**Table 3:** List of key studies of bovine Babesia/babesiosis in Pakistan

Pathogen (s) detected	Host (s)	Location (s)	Study population and sampling design	Sampling period	Detection method (s)	Percentage of test positive animals (proportion)		Reference
						Cattle	Buffaloes	
*Babesia* sp.	Buffaloes, cattle (local breeds)	Karachi	Healthy animals slaughtered in abattoir; convenient	Sep 1984 to Feb 1985	Blood smear	4.2 (4/95)	1.4 (3/219)	[51]
*Babesia* sp.	Buffaloes, cattle (local breeds)	Hyderabad	Healthy animals from local private dairy farms; convenient	Oct 1990 to Dec 1991	Blood smear	1 (1/100)	1 (1/100)	[40]
*Babesia* sp.	Buffaloes, cattle (local and exotic breeds)	Attock, Islamabad	Healthy animals from public livestock farms; convenient	Sep 1999 to May 2001	Blood smear	0.65 (2/307)	0 (0/155)	[41]
*B. bigemina*	Cattle (local breeds)	Peshawar	Healthy animals from local private dairy farms; convenient	2001	Blood smear	^1^ *Bbi*: 1.75 (5/285)	Not applicable (NA)	[42]
*B. bovis*						^2^ *Bbo*: 2.80 (8/285)		
*Babesia* sp.	Cattle (Friesian and Jersey)	Kasur	Healthy animals from a public livestock farm; convenient	Jul 2003 to Jun 2004	Blood smear	Holstein: 2.5 (5/200)	NA	[43]
						Jersey: 2.5 (5/200)		
*B. bigemina*	Cattle (local breeds)	Malakand Agency	Healthy animals from local private dairy farms; convenient	-	Blood smear	5.2 (42/794)	NA	[52]
*Babesia* sp.	Cattle (local and exotic breeds)	Malakand Agency	Healthy animals from local private dairy farms; convenient	-	Blood smear	6.6 (73/1100)	NA	[53]
*B. bigemina*	Friesian cattle	Kasur	Healthy animals from local private dairy farms; convenient	-	Blood smear,	^3^BS: *Bbi* = 6 (6/100); *Bbo* = 3 (3/100)	NA	[7]
*B. bovis*					PCR			
						^4^PCR: *Bbi* = 13 (13/100); *Bbo* = 7 (7/100)		
*Babesia* sp.	Cattle (cross-bred calves)	Sahiwal	Healthy animals from a public livestock and local private dairy farms; convenient	May to Jul 2005	Blood smear	7.2 (30/415)	NA	[54]
*B. bigemina*	Cattle (cross-bred)	Sahiwal	Healthy animals from a public livestock farm; convenient	Jun to Aug 2005	Blood smear, PCR	BS: 18 (18/100)	NA	[49]
*B. bovis*						PCR: 29 (29/100); *Bbi* = 18 (18/100); *Bbo* = 11 (11/100)		
*B. bovis*	Buffaloes, cattle (local breeds)	Bahawalnagar, Bhakar, Layyah, Multan,	Healthy animals from local private dairy farms; convenient	Jan to Aug 2010	Blood smear, PCR	BS: 2.7 (4/144)	PCR: 23.1 (9/39)	[50]
						PCR: 17.1 (18/105)		
		Muzaffar Garh, Vehari						
*B. bovis*	Buffaloes	Karachi	Healthy animals from the Landhi Dairy Colony; convenient	Apr to Oct 2011	Blood smear	N/A	3 (3/100)	[46]
*B. bigemina*	Cattle (local and exotic breeds)	Sargodha	Healthy animals from local private dairy farms; multi-stage cluster random	Aug 2008 to Jul 2009	Blood smear	6.57 (23/350)	NA	[47]
						^5^Mixed: 1.7 (6/350)		
*B. bigemina*	Cattle (local and exotic breeds)	Khushab, Rawalpindi, Sargodha	Healthy animals from local private dairy farms; convenient	Sep 2009 to Aug 2010	Blood smear	4.8 (50/1050)	NA	[48]
*B. bigemina*	Cattle (local breeds)	Charsadda, Swabi	Healthy animals from local private dairy farms; convenient	Jan 2010 to Dec 2011	Blood smear	*Bbi* = 19 (19/100); *Bbo* = 11 (11/100)	NA	[55]
*B. bovis*								

^1^ *Babesia bigemina;* ^2^ *Babesia bovis*; ^3^Blood smear; ^4^Polymerase chain reaction; ^5^Mixed infection of *B. bigemina* with either *A. marginale* or *T. annulata*; − denotes lack of information

#### Anaplasmosis

In Pakistan, bovine anaplasmosis is mainly caused by *A. marginale* [56]; thus far, 12 studies have reported the disease in cattle (*n*umber of districts studied = 10) and water buffaloes (*n* = 8) from the provinces Punjab (number of districts studied = 9), Khyber Pakhtunkhwa (*n* = 2) and Sindh (*n* = 2) (Fig. 1; Table 4). Based on conventional (i.e., stained blood smear) methods, the mean (±standard error of mean) prevalence rates of anaplasmosis in water buffaloes and cattle in Pakistan are 19.2 ± 4.7 % (range: 3.1–60.0 %) and 13.2 ± 3.7 % (range: 4.3–60.0 %), respectively. Haider, and Bilqees [8] reported the highest (60.0 %) prevalence of anaplasmosis in both water buffaloes and cattle in Karachi and Sindh; however, all subsequent studies from Karachi and environs (i.e., Hyderabad) as well as other parts of the country have reported lower prevalences (9–22 %) (Table 4), which appear to be within the expected range for endemic regions. Recently, using the molecular detection methods, Ashraf *et al.* [57] reported a considerably higher prevalence (41.0 %) of *Anaplasma* species. Bovine anaplasmosis has been found to be higher during the summer season (April to September) in exotic breeds of cattle, the Kundi breed of water buffalo, female animals, and small dairy farms [47, 48, 56]. Species of *Hyalomma* and *Rhipicephalus* (ticks) have been linked to the transmission of bovine anaplasmosis in Pakistan; however, no information is available either on experimental transmission of the disease using tick vectors or on *Anaplasma* species in un-engorged, potential tick vectors. Figure 1 and Table 4 show the locations of anaplasmosis, according to studies conducted thus far in Pakistan; almost all of these studies were carried out around major veterinary research institutions or near public livestock research stations, suggesting a sampling bias. Furthermore, convenient sampling seems to be another source of bias in all published studies, except those by Ali *et al.* [48] and Sajid *et al.* [56] who specified the sampling design of their study (see Table 4).

**Table 4:** List of key studies of bovine Anaplasma/anaplasmosis in Pakistan

Pathogen (s) detected	Host (s)	Location (s)	Study population and sampling design	Sampling period	Detection method (s)	Percentage of test positive animals (proportion)		Reference
						Cattle	Buffaloes	
*Anaplasma marginale*	Buffaloes, cattle (local breeds)	Karachi	Animals slaughtered in abattoir; convenient	Nov 1984 to Dec 1985	Blood smear	60 (30/50)	60 (60/100)	[8]
*A. marginale*	Buffaloes, cattle (local breeds)	Hyderabad	Healthy animals from local private dairy farms; convenient	Oct 1990 to Dec 1991	Blood smear	^1^ *Am*: 11 (11/100)	*Am*: 19 (19/100)	[40]
*A. centrale*						^2^ *Ac*: 7 (7/100)	*Ac*: 11 (11/100)	
*A. marginale*	Buffaloes, cattle (local and exotic breeds)	Attock, Islamabad	Healthy animals from public livestock farms; convenient	Sep 1999 to May 2001	Blood smear	17.3 (53/307)	12.9 (20/155)	[41]
*A. marginale*	Cattle (local breeds)	Peshawar	Healthy animals from local private dairy farms; convenient	2001	Blood smear	*Am*: 4.2 (12/285)	Not applicable (NA)	[42]
*A. centrale*						*Ac*: 3.86 (11/285)		
						^3^Mixed: 4.21 (12/285)		
*A. marginale*	Buffaloes, cattle (local breeds)	Hyderabad	Healthy animals from local private dairy farms; convenient	Feb to Apr 2004	Blood smear	*Am*: 22 (55/250)	*Am*: 13.6 (34/250)	[58]
*A. centrale*						*Ac*: 9.2 (23/250)	*Ac*: 8.4 (21/250)	
						Mixed: 20.8 (52/250)	Mixed: 8 (20/250)	
*A. marginale*	Buffaloes, cattle	Different districts of Khyber Pakhtunkhwa	Healthy animals from local private dairy farms; convenient	Jun to Jul 2003	Blood smear	15.1 (8/53)	26.1 (17/65)	[59]
*A. marginale*	Cattle (local and exotic breeds)	Sargodha	Healthy animals from local private dairy farms; multi-stage cluster random	Aug 2008 to Jul 2009	Blood smear	9.7 (34/350)	N/A	[47]
						^4^Mixed: 3.1 (11/350)		
*A. marginale*	Cattle (local and exotic breeds)	Khushab, Rawalpindi, Sargodha	Healthy animals from local private dairy farms; convenient	Sep 2009 to Aug 2010	Blood smear	5.8 (61/1050)	N/A	[48]
*A. marginale*	Buffaloes	Karachi	Healthy animals from the Landhi Dairy Colony; convenient	Apr to Oct 2011	Blood smear	N/A	9 (9/100)	[46]
*Anaplasma* sp.	Buffaloes	Bahawalnagar, Burewala, Kohat, Layyah, Multan, Peshawar	Healthy animals from local private dairy farms; random	May to Sep 2001	^5^PCR-RFLP	NA	*Anaplasma* sp.	[57]
*A. marginale*							41 (115/281)	
							*Am*: 17 (20/155)	
*A. marginale*	Cattle (local and exotic breeds)	Khushab, Rawalpindi, Sargodha	Healthy animals from local private dairy farms; convenient	Sep 2009 to Aug 2010	Serology - ^6^ELISA	-	N/A	[60]
*Anaplasma* sp.	Buffaloes, cattle (local and exotic breeds)	Khanewal	Healthy animals from local private dairy farms; simple random	May 2011 to April 2012	Blood smear	4.1 (34/836)	4.29 (30/700)	[56]

^1^ *Anaplasma marginale*; ^2^ *Anaplasma centrale*; ^3^Mixed infection of *A. marginale* and *A. centrale*; ^4^Mixed infection of *A. marginale* with either *T. annulata* or *B. bigemina*; ^5^Polymerase chain reaction-restriction fragment length polymorphism; ^6^Enzyme-linked immunosorbent assay; − denotes lack of information

### Methods currently used in Pakistan for the diagnosis of TBDs

Presently, a number of approaches are being used for the diagnosis of TBDs and for studying their epidemiology. For example, clinical signs, and the detection of pathogens on stained blood smears (e.g., demonstration of *Anaplasma* spp. as inclusion-bodies within erythrocytes, *Babesia,* and *Theileria* spp. as piroplasms within erythrocytes, and *Theileria* spp. as schizonts within leucocytes) or smears of lymph node biopsies (for *Theileria* spp.) have been the most commonly used diagnostic methods (see Tables 2,3 and 4). In addition, a number of serological tests have been used in epidemiological surveys of different TBDs; they include a competitive enzyme-linked immunosorbent assay (cELISA) using the MSP-5 antigen [61] and an indirect immunofluorescence antibody technique (IFAT; [62]) for *Anaplasma* spp., and ELISA, and IFAT for *Babesia* spp. [18] as well as *Theileria* spp. [63, 64]. Although various polymerase chain reaction (PCR) techniques have been developed for anaplasmosis, babesiosis [61, 65–68] and tropical theileriosis [69–71] in different laboratories around the world, these methods are not yet routinely used for surveys in developing countries such as Pakistan, possibly due to the relatively high cost of reagents and/or the expertise required to perform these assays.

In Pakistan, almost all of the studies reporting bovine TBDs have used the stained blood smear as a diagnostic method (see Tables 2,3 and 4), and only a few studies, in recent years, have utilized PCR for the detection of *T. annulata* [7, 32, 35–37], *Babesia* spp. [7, 49, 50] and *Anaplasma* spp. [57]. In addition, Atif *et al.* [60] used, for the first time, a serological assay to estimate the prevalence of *Anaplasma* infection or exposure in cattle in Pakistan using the MSP-5 cELISA, although, surprisingly, these authors did not mention the overall seroprevalence in their paper. In addition to the use of conventional and modern diagnostic methods, a number of studies reported the haematological and biochemical status of water buffaloes and cattle clinically affected by theileriosis, babesiosis, and anaplasmosis [36, 37, 44, 45, 48, 52, 53, 72, 73]. For instance, Durrani *et al.* [44] reported a significant decrease in packed cell volume (PCV), total erythrocyte count (TEC), and haemoglobin (Hb) concentration in water buffaloes suffering from tropical theileriosis. Similarly, Qayyum *et al.* [45] studied the haematological profiles in exotic and cross-bred cattle with clinical signs consistent with theileriosis, and found a significant decrease in the mean values of PCV, TEC, Hb, and total leukocyte count (TLC) in diseased compared with healthy cattle. Recently, Khan *et al.* [73] investigated haematological and biochemical changes associated with bovine theileriosis in cross-bred cows, and found significant changes in concentrations of total serum protein, globulins, albumin, phosphorus, calcium, triglycerides, cholesterol, alanine transaminase activity, and serum bilirubin.

### Prevention and control of TBDs in Pakistan

Currently, a number of methods, including chemical tick control, chemotherapy as well as prophylaxis and/or vaccination are used worldwide to reduce economic losses resulting from TBDs in bovines [74]. Although tick control has been the most commonly used method of controlling these TBDs, the reliance on chemicals is decreasing due to the possible emergence of acaricide-resistant ticks [75] as well as public health concerns about residues in meat and milk [76, 77].

A number of studies have tested the efficacy of various drugs (individually or in combination with antibiotics) for the treatment or prophylaxis of theileriosis, babesiosis, and anaplasmosis using buparvaquone, diminazene aceturate + imidocarb dipropionate and oxytetracycline, respectively [38, 43, 45, 54, 78]. Owing to limited understanding of bovine TBDs, currently, no schedule for the chemoprophylaxis of these is in place in Pakistan. For tick control, grooming is the most commonly used strategy, particularly at small-holding farms. In Pakistan, grooming involves the manual removal of ticks and burning them on the fire made with cattle dung cakes, which is also a common tick control strategy in other developing countries [79, 80]. Another method to treat and/or control ticks is spraying the animals and their surroundings with cypermethrine during high-risk months (May to September) of the year. Recently, with the establishment of large commercial dairy farms, a number of acaricides, such as macrocyclic lactones (Tariq Abbas, personal communications), are also being used to control ticks on water buffaloes and cattle in Pakistan.

## Conclusions and future scope

Most previous studies of bovine anaplasmosis, babesiosis, and theileriosis in Pakistan have (i) estimated the prevalence of these TBDs in one or more districts around major veterinary research institutions or on public livestock research stations using conventional diagnostic methods, (ii) evaluated the efficacy of various drugs against these diseases, or (iii) assessed changes in haematological and biochemical parameters in water buffaloes and cattle affected by these three TBDs. Although epidemiological studies have provided some insight, the interpretations from various investigations are compromised because of limitations in study design and the diagnostic methods employed. On one hand, some studies did not consider agro-ecological zones, production system, age structure of the bovine population, sampling strategy and season, or breed, which are all factors that can affect the prevalence of TBDs. On the other hand, in most cases, molecular methods were not used to achieve a genetic identification of the species or genotypes of pathogens present. Therefore, this review indicates that a lack of accurate data on the epidemiology of bovine TBDs and their vectors makes it challenging to assess their economic impact on water buffaloes and cattle production in the different agro-ecological zones of this country.

Given that the dairy industry is rapidly expanding, nationwide epidemiological surveys should be carried to establish the spatial distribution and economic impact of TBDs and ticks, to guide future research, and control. As published information relates to cattle, future work should focus on estimating the disease impact on the water buffalo, which is the mainstay dairy animal in Pakistan. It will also be important to assess whether water buffaloes and/or other animals (e.g., wildlife) are reservoir hosts for pathogens that are transmissible to cattle, given that *T. parva* infection is known to be asymptomatic in the African buffalo (*Syncerus caffer*) but causes disease in cattle, leading to significant morbidity, and mortality [81]. Furthermore, as multi-host pathogens (including ticks) are economically important and can cross-transmit between domesticated and wild animals [82], improved control strategies for bovine TBDs of livestock in Pakistan will need to consider findings from future surveys of wildlife for ticks and blood-borne pathogens.

Although various studies have attempted to genetically characterize *T. annulata* [7, 32, 36, 37], *Babesia* spp. [7, 49, 50] and *Anaplasma* spp. [57] based on the presence or size of products produced by PCR, amplicons were not sequenced to verify their specificity and genetic identity. To date, only Khan *et al.* [35] sequenced nuclear ribosomal DNA (18S and internal transcribed spacer, ITS) regions from *T. annulata* isolates from cattle from two districts in Punjab province; these authors found that some of the *T. annulata* sequences matched those from Turkey, while others were novel, suggesting a genetic distinctiveness. Therefore, there is a need to focus on exploring the genetic composition of tick-borne pathogens of bovines in Pakistan and assessing disease transmission patterns.

The present review shows clearly that the challenge of studying TBDs in Pakistan relates largely to the limitations of current methods used for the diagnosis of infections and disease. Before large-scale field studies of the epidemiology of TBDs can be undertaken, it will be essential to be able to accurately diagnose, identify, and differentiate the respective pathogens. For future epidemiological studies, it is recommended that specific and sensitive PCR-based tools, such as real-time, or multiplex-tandem PCR [83–86] and/or single-strand conformation polymorphism (SSCP) analysis [87, 88], be used for diagnostic, systematic, and population genetic studies, thereby helping to identify species and genotypes of pathogens.

These or similar tools might be used to specifically explore oriental theileriosis in Pakistan. Although *T. orientalis* has been detected in India [89] and Sri Lanka [90], it appears not to have been studied or reported from Pakistan. It is possible that autochtonous or introduced infections of *T. orientalis* occur in bovines in this country. For instance, thousands of dairy cattle are imported to Pakistan from the State of Victoria in Australia. Given that there is evidence of recent outbreaks of oriental theileriosis in this state, that *T. orientalis* is now endemic there [91–93] and that blood samples from cattle are not screened using molecular tools for piroplasms prior to export to Pakistan, it would be important to estimate the prevalence of *T. orientalis* genotypes and their intensity of infection in dairy cattle upon arrival to Pakistan, and, if found, then to track these genotypes and assess whether they spread to local breeds of cattle, water buffaloes and/or other livestock or wildlife.

Although the current control of bovine TBDs in Pakistan relies mainly on tick control using acaricides, no study has yet assessed the status of acaricidal resistance in ticks, as such resistance has been recorded in various countries [75]. In terms of alternative methods of tick and TBD control, once the epidemiological status of the three main bovine TBDs in Pakistan has been studied in some detail, it would be useful to consider assessing the utility of vaccines. Vaccines are available to protect cattle against bovine theileriosis, babesiosis, and anaplasmosis [94]. For instance, a vaccine containing *in vitro*-attenuated *T. annulata*, and developed in Israel, is currently being used to control tropical theileriosis in China, Iran, Israel, Morocco, Tunisia, and Turkey [94]. Similarly, a live vaccine containing attenuated strains of *B. bovis* and *B. bigemina* and *A. marginale* was developed in Australia, and has been widely used for the prevention/control babesiosis and anaplasmosis in Argentina, Australia, Israel, South Africa, and some South American countries [94]. As the large-scale production of such vaccines under good manufacturing practice (GMP) is costly, vaccines against local pathogen species and strains might be established manufactured, marketed, and sold by government-funded institutions in Pakistan, to ultimately achieve improved, and sustainable control of TBDs in the longer term.

Another possible vaccination strategy to control TBDs could be the use of tick vaccines, which might offer a cost-effective, environmentally friendly alternative to the use of acaricides. For example, the tick vaccine for cattle based on the *R. microplus* Bm86 gut-antigen (TickGARD®) has proved to be effective in Australia and Cuba [94]. Similarly, a vaccine against *H. anatolicum*, a three-host tick vector transmitting *T. annulata*, could also be another alternative [95]. However, the major disadvantage of using tick vaccines is that they will not confer protection against multiple tick species. Nevertheless, the use of a tick vaccine can reduce the acaricidal treatments and a reduction in TBDs [96]. Another alternative strategy that could be explored to control ticks and TBDs in bovines in Pakistan, is the development of tick-resistant breeds of cattle. This can be done by crossing the local Pakistani cattle breeds (*Bos indicus*; relatively resistant to ticks) with high milk-producing exotic cattle breeds (*Bos taurus*; susceptible to tick infestation) as some studies have noted milk yields in cross-bred animals appear not to be compromised [97, 98].
